# The Medical Freshman

**Published:** 1889-09-14

**Authors:** 


### The Hospital

A Weekly Institutional Journal of

Science, Medicine, Nursing, and Philanthropy.

*For Hospitals, Asylums, Medical Practitioners, Students, Nurses, Families, Parishes, Congregations, and the Charitable Public.*

Vol. VI. No. 155.] [September 14, 1889.

### The Medical Freshman.

On the first of October in England, and about the last day of the same month in Scotland, some hundreds of young men, having duly passed their preliminary examinations, will register themselves, as by law prescribed, and enter the hospital or college hall for the first time as medical students. To most of them it is a proud moment; to some few a day of nervous apprehension. But the occasions which really try the nerves of embryo doctors are the first visit to the dissecting-room and attendance on the first serious surgical operation. Undoubtedly it is a gruesome moment for a youth of eighteen when he opens the dissecting-room door for the first time and sees arranged in order round the room ten, fifteen, or more solid tables on each of which is placed a dead man's body, all stark and naked, waiting for the dissector's knife. Few young men enter without a painful sinking of the heart, and fewer still commence work on their own "parts," without feeling a sickening sensation as of dread and horror. The feeling soon wears off. At the end of the first week the dissecting-room is as the lecture-room, or even as the library at home. Young men are as blithe and cheerful there as in the cricket-field or on the waxed floor.

And yet the dissecting-room is not quite the same as any other place in the world; nor is the calling of the doctor, to which it is the entrance and the portal, quite the same as any other calling. The near and intimate knowledge which the medical student seeks and acquires of all the secret places of the human body well prefigures the near and intimate relations he will sustain towards individuals, families, and communities of men and women in his later life. It is one thing to bake bread which is to be delivered at a customer's door, or to make steam-engines which may drag through space all sorts of men and women whom the maker will never even see; but it is quite another to go to a patient's house, to enter as of right, to see him in the privacy of his bedchamber, to examine his body in the minutest way, to ask him the most searching questions, to hear his most intimate and private confidences—confidences which he does not and cannot give to any other person living, man or woman—to do this day by day and hour by hour for a lifetime; that, we say, is quite a different thing from any ordinary trade or occupation whatsoever. A calling like this demands very peculiar qualities in the men who follow it; and there is no doubt that if the great world could have its way it would select very peculiar persons for it. The great world cannot have its way, and it is undoubtedly well that it cannot. As all sorts and conditions of men go to the making of the world, so it is well that all sorts and conditions of men should know the secrets of anatomy and medical practice, and that the world should see all sorts of men handling the tools of the doctor, and using his peculiar knowledge in their own peculiar way.

But though all sorts of men may and should enter the portals of medicine and learn the secrets alike of the dead and of the living, there is an obligation laid upon each to open his mind and to take in receptively the peculiar influences of the environment in which all medical students have voluntarily chosen to place themselves. The man who can help to fight a patriot's victorious battle without enthusiasm, who can listen to the divinest strains of music with no stirrings of rapture, who can stand by the deathbed of parent or child with no agonised questionings of the possibilities of an immortal life, may possess the human form and even the human brain; but he is not what any poet or man of science would select as a typical specimen of the highest order of the primates. So also, that medical student who can study the structure and functions of the human body by means of a now dead, but once living man, who can gradually enter into the most secret places of knowledge of the living as well as of the dead, who can witness the pains and agonies, the ruin and destruction, which accompany the often tragic passage of men and women through this brief and incomprehensible life—the student who can do this and remain uninfluenced and untaught by it, must be a man of stupidity more dense than the hopeless idiot's, of coarseness more coarse than that of the barbarian or the brute.

The youth who becomes a student of medicine thereby places himself in a scientific environment. But what is a scientific environment? It is a school in which Nature opens many of her wonderful mysteries and ordered secrets to the open and enquiring mind. A rare, patient, and intelligent teacher is Nature, with her assistants and demonstrators whom the world dubs "learned professors." To the dull and stupid student the professors are the only teachers; and very exacting, imperious, and unsympathetic teachers the lazy and the incompetent consider those professors to be. In truth, they are, as a rule, both patient, considerate, and helpful, if the student will but be docile and diligent. But Nature herself is the teacher of teachers. The true student is grateful to the professor for holding the candle for him, and for showing him the way to and through the sometimes dark corners and difficult paths he has to traverse. But he always feels that he must see everything with his own eyes, as well as through the professor's spectacles; that he must handle everything which can be handled, with his own hands; that he must seize upon and hold as his own permanent possession and property all knowledge which lies open to his view. It is one of the most noble characteristics of knowledge that its possessor can give it away to others and be none the poorer, but rather the richer himself for so doing. The basest of all misers is he who possesses knowledge, but does not like to impart it. Such misers are practically unknown in the hospital and the medical school.

But a scientific environment does not merely enrich a man with knowledge. It shapes him as the skilled worker in clay shapes his vases and his urns; it inspires him as the gentle breath of summer fills the leaves with murmuring music, or as the pulses of the spring inspire the birds with song. It gives him both form and life. Whereas he entered his new environment as a commonplace and undifferentiated schoolboy, he shall find himself, after years of fashioning, if he have but a plastic soul, a "man of science." And what is a true man of science? A learned pedant, proud of his knowledge and scornful of his fellow-creatures? Nay, but a docile, modest, open-eyed, reasonable, patient, and sympathetic man. Do some men of science fail to answer to this description? Do they blush to find themselves so little resembling it? Let us hope they do. But the Faradays, the Darwins, the Newtons, the Bacons, and the Aristotles were men of this kind, and not a few now living are undoubtedly of the same rare and noble type. Of course, nobody can make a silk purse out of a sow's ear. A sow's ear is a sow's ear, and however much it may be improved by being cut off and boiled until it is tender enough for eating, or tanned until it is turned into a rather poor kind of leather, it is still in fact and essence a sow's ear. Let nobody therefore be surprised that the environment of a medical education leaves many young men but little changed in character and form. But where there is a young man with true mental capacity, genuine breadth and expansiveness of mind, docility of temper, and steadfast industry, there is a young man whom the environment of one of the best medical schools will fashion into a true man of science in due time.

But the medical student must become a specialist as well as a man of science. He must learn his business. That business is often of a very homely kind, though it cannot be well done by an intellect that is merely homely. The true basis of practice is not homely guessing and routine, but accurate knowledge of facts and natural processes. The physician's prescription may contain nothing but rhubarb and soda, but the operations of his mind that finally end in the rhubarb and soda often deal with subjects as profound and more difficult than the mathematician's subtlest calculations, or the metaphysician's keenest and most elaborate reasonings. There are two kinds of doctors in the world, and, practically two only, the reasoner, and the man who works by "rule of thumb." There is no more worthless or despicable fellow in the world than the "rule-of-thumb" doctor. It is he who has brought upon the profession all the discredit and ignominy which have covered it in past ages, and which still cling to it in many men's minds. A man had better be a trumpet-blower for an infallible ointment or a sandwich man for a quack pill than a "rule-of-thumb" practitioner of medicine. He who practices his profession so may be sure of at least two things—that he will succeed in killing a good many of his patients in this world, and of finally destroying his own worthless self in the next. The true physician is first a man of intelligent science, and then a man of plain, timely, and resolute practice; doing, with his own hands, when necessary, everything he knows or can discover for his patients' comfort and quick restoration to health. Every medical student who has formed the resolution to become such a man has entered upon a calling second in nobility to none in the world.

